# Non-Destructive Testing of Steel Corrosion Fluctuation Parameters Based on Spontaneous Magnetic Flux Leakage and Its Relationship with Steel Bar Diameter

**DOI:** 10.3390/ma12244116

**Published:** 2019-12-09

**Authors:** Qingyuan Zhao, Jianting Zhou, Qianwen Xia, Senhua Zhang, Hong Zhang

**Affiliations:** 1 State Key Laboratory of Mountain Bridge and Tunnel Engineering, Chongqing Jiaotong University, Chongqing 400074, China; zhaoqingyuan@mails.cqjtu.edu.cn (Q.Z.); jtzhou@cqjtu.edu.cn (J.Z.); xiaqianwen1122@163.com (Q.X.); shzhang@mails.cqjtu.edu.cn (S.Z.); 2 College of Civil Engineering, Chongqing Jiaotong University, Chongqing 400074, China

**Keywords:** spontaneous magnetic flux leakage, spontaneous magnetic flux leakage, non-destructive testing, steel bar outer cladding, steel bar corrosion

## Abstract

In an actual structure, the arrangement of steel bars is complicated, there are many factors affecting the corrosion of steel bars, and these factors affect each other. However, accurately reflecting the corrosion of steel bars in actual engineering through theoretical calculations is difficult. Besides, it is impossible to detect and evaluate steel bars rust completely and accurately. This article is based on spontaneous magnetic leakage detection technology and adopts the method of stage corrosion and scanning along the reinforcing bar. Based on spontaneous magnetic flux leakage detection technology, the linear change rate of the tangential component curve of the magnetic flux leakage signal generated after the corrosion of a steel bar is studied, and a comparison is made between the steel bar coated concrete samples with different steel bar diameters. In this paper, the “origin of magnetic flux leakage signal” is defined as a reference point, which is convenient for effectively comparing the magnetic signal curves under all operating conditions. Besides, the “rust-magnetic fluctuation parameter” is proposed to accurately reflect the sudden change of leakage magnetic field caused by disconnection due to the corrosion of a steel bar. A new data processing method is provided for the non-destructive testing of steel corrosion using the spontaneous magnetic flux leakage effect, which can effectively reduce the influence of steel bar diameter on magnetic flux leakage signal and improve the precision of non-destructive testing technology of steel bar corrosion using the metal magnetic memory effect.

## 1. Introduction

Reinforced concrete is one of the most important elements in contemporary structures and can directly determine tensile, shear, seismic, and impact resistance. Structural performance is damaged by rebar rust inside the reinforced concrete structure, affecting its durability and safety and leading to sudden disasters. Therefore, effective detection of rust concrete structure corrosion is the key to ensuring the safety of in-service structure operation [1,2,3].

The corrosion of steel bars inside reinforced concrete structures can be roughly detected by existing inspection technology [4,5]. In previous studies, we learned to determine the extent of the rusted area using the intersection points of the magnetic flux leakage signal curves with different lift-off heights. However, the accuracy of this detection is seriously reduced due to the generation of magnetic flux leakage signals, the influencing factors in the acquisition process, and cross-effects. A method for faster and more accurately detect the corrosion of steel bars inside reinforced concrete structures has become a major concern of researchers around the world.

The corrosion parameters of a steel bar and the defects of internal steel bars are directly detected by breakage detection technology, which strips the concrete surface of the reinforced concrete structure [6]. Although the method is accurate and intuitive, it is time-consuming and labor-intensive, the structure is damaged, and maintenance is difficult, causing different degrees of secondary damage to the structure. Therefore, this has a more serious impact on the durability and safety of the structure. Damage detection is difficult to implement in various cases, such as in the main load-bearing parts of buildings or important bridge structures and their critical parts. Non-destructive testing techniques include electrochemical methods, analytical methods, and physical methods. Among them, the electrochemical method includes the half battery method. This method detects the potential of each part relative to the standard half-cell from the surface of the structure and then makes a potential map, resulting in determining the corrosion of the steel bar. However, it cannot accurately determine the true degree of corrosion [7]. The linear polarization method requires a small current to be applied to the steel bar, and only the rust area of the steel bar surface can be calculated. However, the rust depth cannot be calculated [8,9]. A constant current pulse is applied to the steel bar in the constant current pulse method, which is an upgraded version of the linear polarization method, but the detection efficiency can only be improved, not the detection accuracy [10]. AC impedance spectroscopy can reflect more comprehensive information in the rust concrete corrosion system, but it is less used in engineering tests due to long test time, difficult analysis, and expensive test equipment [11]. The on-site measurement is required in analysis method to affect the internal and external factors of steel corrosion and corrosion, including the thickness and strength of the concrete protective layer, the diameter of the steel bar, the longitudinal crack width of the structure, the depth and content of the harmful ion immersion structure, and the environmental conditions of the structure, in order to infer the degree of corrosion of the steel inside. The disadvantage of this method is that there are too many factors that need to be considered and it is cumbersome to calculate [12]. The physical method mainly includes the ultrasonic testing method, which is troublesome in operation and requires high flatness of the concrete surface [13,14]. The X-ray detection method is expensive; besides, the radiation generated by X-rays causes irreversible damage to the human body [15,16]. The eddy current testing method detects shallow depth and is not suitable for large structures [17,18]. Infrared thermography requires different temperatures and is highly susceptible to the environment [19,20]. Moreover, acoustic emission technology needs external sound waves to be applied to the structure, which is cumbersome to operate [21,22]. These methods are the most popular non-destructive testing techniques and they all have certain deficiencies; furthermore, these technologies can only detect the corrosion of steel bars to a certain extent and cannot accurately detect the rust degree.

As a ferromagnetic material, the steel bar is magnetized to form an initial magnetic field under the action of the earth’s magnetic field. When the steel bars are rusted, the magnetic domain structure orientation and irreversible reorientation of magnetostrictive properties occur in the rust position of the steel bars under the joint action of rust and a geomagnetic field. Simultaneously, the fixed nodes of the magnetic domains appear in these regions, and the magnetic poles are generated to form the demagnetizing field. Therefore, the magnetic permeability of the rusted areas of the steel bars is minimized, forming a leakage magnetic field on the surface. This phenomenon is called spontaneous magnetic leakage effect; the magnetic flux leakage signal is passed. Collection and analysis can obtain the result of steel corrosion [23,24,25].

Compared with other non-destructive testing methods, the use of spontaneous magnetic flux leakage effects has great advantages in the non-destructive testing of steel corrosion, as follows. (1) It is not necessary to apply an additional magnetic field to the reinforced concrete structure, and the geomagnetic field is only used as a natural excitation source, facilitating operation, reducing cost, and eliminating the influence of the applied magnetic field error on the detection result. (2) The magnetic sensor is used to collect the magnetic flux leakage signal on the surface of the reinforced concrete structure to analyze the corrosion of the steel; its accuracy and sensitivity are much higher compared to other non-destructive testing techniques [26].

At present, the non-destructive testing technology for detecting the corrosion of steel bars in reinforced concrete structures using the spontaneous magnetic flux leakage effect has been able to achieve a good qualitative analysis. The further refinement of this technology remains to be further studied. In this paper, reinforced concrete specimens are used as research objects; the electrochemical corrosion principle is applied to accelerate the stage rust. The magnetic signal probe is employed to collect the magnetic flux leakage signals generated after the corrosion of the test pieces. Besides, the data of each group are compared, analyzed and discussed. The “origin of magnetic flux leakage signal” is defined as the reference point for all magnetic signal curves. The “rust magnetic fluctuation parameter” is proposed to more accurately link the corrosion of the steel bar with the change of the magnetic flux leakage signal. Moreover, take the influencing factor in the detection of the steel bar diameter as the main research object. The relationship between the change of the magnetic flux leakage signal and the diameter of the reinforcement with the deepening of the corrosion of the reinforcement was accurately verified.

## 2. Materials and Methods

### 2.1. Test Preparation

**Figure 1:**
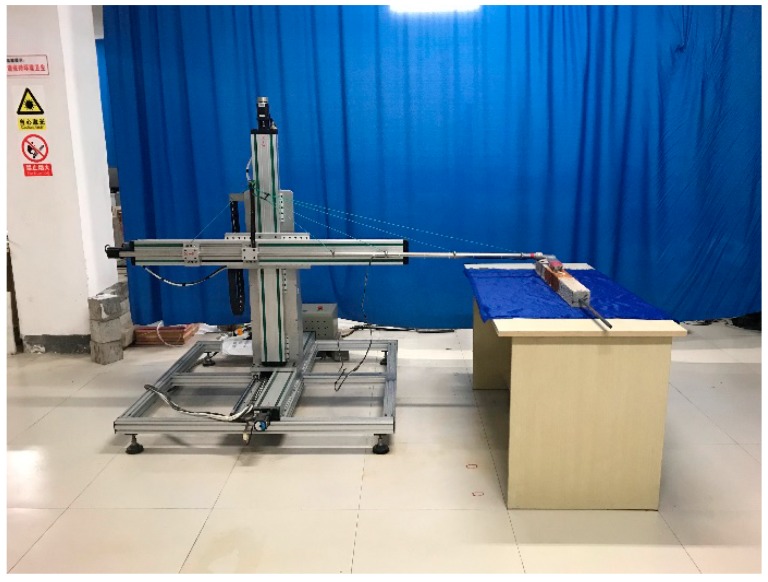
Automatic three-axis magnetic signal testing device.

The test pieces used in this test are HRB400 threaded steel bars commonly applied in civil engineering. The lengths of steel bars are 1500 mm and 2000 mm, and the chemical composition is shown in Table 1. A total of two diameters of steel is used in the test; the sizes are 12 mm and 16 mm (Φ12 and Φ16); 8000 mm concrete covering length and three concrete protective layer thicknesses of 30 mm, 40 mm, and 50 mm are used, with two rust widths (b = 100 mm, a = 150 mm). According to the above parameters, a combination of 24 test piece were obtained according to the permutation and combination method—that is, the name of the test piece is its size parameter combination. The test piece number and name are shown in Table 2. The test instrument used to accelerate the corrosion of steel-reinforced concrete specimens is the ATS3005S-3D dual DC power supply of ATTEN (Nanjing, China). The magnetic flux leakage signal generated after the corrosion of the steel-reinforced concrete specimen is collected by the self-designed and manufactured three-axis spontaneous magnetic flux leakage signal acquisition device. As shown in Figure 1, the device can continuously collect and record the magnetic flux leakage signals in the *x*, *y*, and *z* directions. The magnetic signal probe of the device is the Honeywell HMR2300 magnetic flux leakage signal collector which range is ±2 Gauss, and the resolution is 67 μGauss.

### 2.2. Experimental Procedure

#### 2.2.1. Electrochemical Acceleration of Corrosion

**Figure 2:**
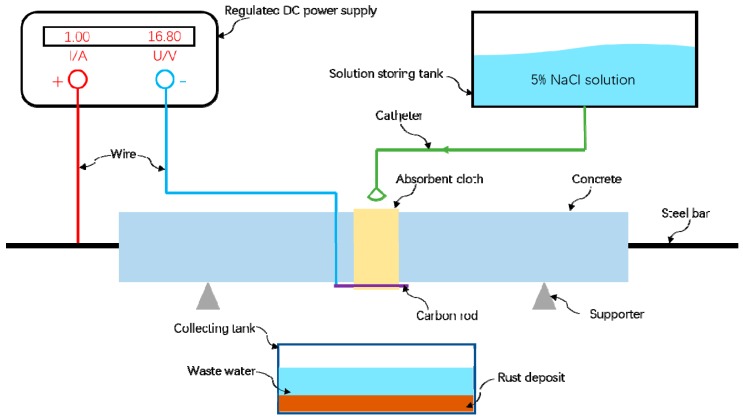
Schematic diagram of rusting of steel-reinforced concrete specimens.

The test consists of two parts. The first part is the stage rust test of a 1.0 A constant current on the steel-reinforced concrete. The electrolyte is infiltrated by the drip irrigation method to facilitate the transmission of electrons and ions using the electrochemical corrosion method. Therefore, the electrochemical accelerated corrosion device shown in Figure 2 was designed. During the process of rusting, the steel bar is connected to the positive electrode of the power source as the anode. The oxidation reaction occurs to lose the cross section of the steel bar. In this test, the 100 mm or 150 mm range of the steel-reinforced concrete test piece is set as the target rust area, and the target of the test piece is wrapped with a strong water-absorbent towel. On the surface of the rusted area, the carbon rod is placed in a towel and connected to the negative pole of the power source to make a cathode. Besides, the electrolyte is a 5% NaCl solution, which is continuously dripped from a high place to the top of the absorbent towel by a dropper. Then, it is infiltrated into the concrete, connecting the whole system into a closed circuit to realize the accelerated corrosion of the steel bar of the steel-reinforced concrete specimen. The waste electrolyte and the rusted product that has leaked out is collected by the container under the test piece. The rust current of each beam was 1.0 A and was kept constant. After 12 h of corrosion, the corrosion condition and crack development of the test piece were suspended, the magnetic signal around it was collected, and then, the next rust was continued.

**Figure 3:**
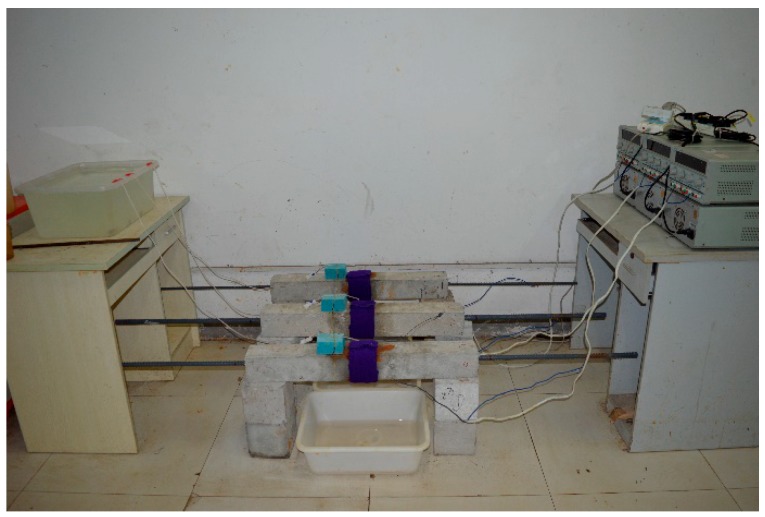
Photograph of the test piece rust test.

The field photos of the corrosion test of reinforced concrete specimens are shown in Figure 3.

During the test piece rusting process, the target rusting area can be in good contact with both the electrolyte and the air, which makes the development mode of rusting similar to natural rusting.

#### 2.2.2. Magnetic Flux Leakage Signal Acquisition

**Figure 4:**
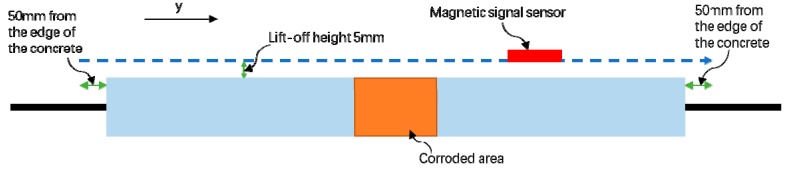
Schematic diagram of the scan path.

The second part is to collect the magnetic signal of the steel-reinforced concrete specimen using the self-designed and manufactured three-axis spontaneous magnetic flux leakage signal acquisition device. Because the geomagnetic field will affect the strength of the magnetic flux leakage signal of the steel bar, the test piece is placed perpendicular to the direction of the geomagnetic field. In addition, the magnetic flux leakage signal component *B_y_* perpendicular to the geomagnetic field direction is collected along the *y*-axis direction perpendicular to the geomagnetic field, which can effectively reduce the influence of the geomagnetic field on the magnetic flux leakage signal during acquisition. As shown in Figure 4, in this part of the test, the magnetic signal probe for the spontaneous magnetic flux leakage signal acquisition height is set to 5 mm. In order to avoid the effect of the end of the steel bar, it moves along the steel bar axial direction at a speed of 500 mm per minute between the 50 mm position outside the concrete portion of the test piece. The magnetic flux leakage signal on the surface of the test piece is continuously collected at a collection speed of once every 2 s; thus, this part of the test is also called as “scanning along the reinforcing bar”. Before the test piece is rusted, the first magnetic signal is collected by the scanning device to obtain the initial magnetic signal of the test piece. After each rusting condition, a magnetic flux leakage signal is collected and compared with the magnetic signal collected for the first time.

### 2.3. Magnetic Dipole Model

**Figure 5:**
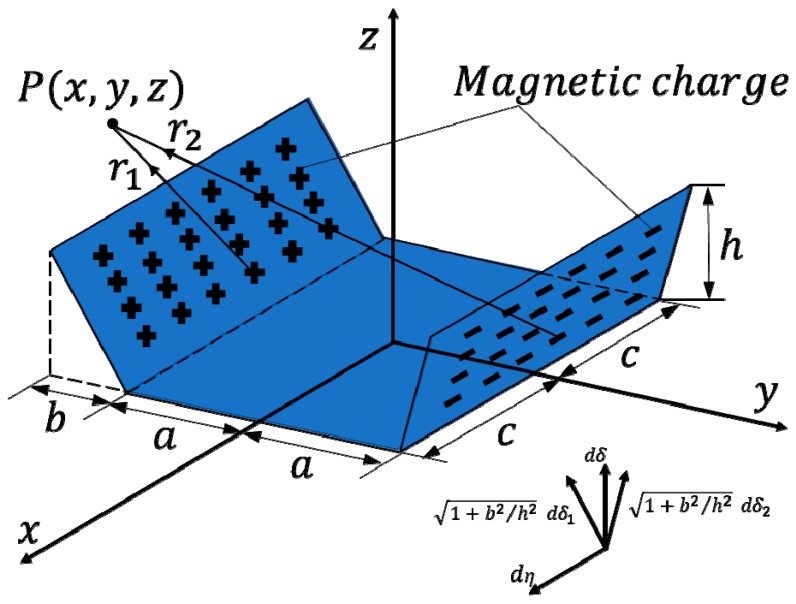
Schematic diagram of the notched magnetic dipole model of the trapezoidal slot.

As shown in Figure 5, the notch is set as a trapezoidal notch for the calculation. The length of the bottom end and the top end of the notch are *2c* in the *x* direction; the width of the bottom end of the notch is *2a* in the *y* direction; the width of the top of the slot in the *y* direction is *2a* + *2b*; the depth of the notch is *h* in the *z* direction.

Two magnetic charge surfaces on the two sidewalls of the trapezoidal slot are formed by the magnetization; the surface magnetic charges of the opposite polarity are uniformly distributed on the magnetic load surface. It is assumed that there is no magnetic charge distribution in the notch and other parts, as shown in Figure 5. Then, the left and right groove walls are centered on the depth *η* in the *x* direction and the height *δ* in the *z* direction; the length in the *x* direction is *dη*; the height in the *z* direction is *dδ*; the width in the side wall direction is (*b*^2^ + *h*^2^)^−2^/*hdδ*; the magnetic field surface charge is denoted by *ρ_ms_*; vacuum magnetic permeability is *μ*_0_ = 4π × 10^−7^ H/m; the magnetic field strength generated by the magnetic charges of these two surfaces at any point in space *P* (*x*, *y*, *z*) is shown in Equation (1):

(1) $$\left\{ \begin{array}{l} d{\vec{B}}_{1}=\left[\begin{array}{l} dB_{1x}\\ dB_{1y}\\ dB_{1z} \end{array}\right]=\frac{\rho_{ms}ds}{2\pi \mu_{0}r_{1}^{3}}{\vec{r}}_{1}=\frac{\rho_{ms}\sqrt{1+b^{2}/h^{2}}d\delta d\eta }{2\pi \mu_{0}\left[{\left(\left|x-\eta \right|\right)}^{3}+{\left(y+a+b-\delta b/h\right)}^{3}+{\left(z-\delta \right)}^{3}\right]}\left(\begin{array}{c} \left|x-\eta \right|\\ y+a+b-\delta b/h\\ z-\delta \end{array}\right)\\ d{\vec{B}}_{2}=\left[\begin{array}{l} dB_{2x}\\ dB_{2y}\\ dB_{2z} \end{array}\right]=-\frac{\rho_{ms}ds}{2\pi \mu_{0}r_{1}^{3}}{\vec{r}}_{2}=-\frac{\rho_{ms}\sqrt{1+b^{2}/h^{2}}d\delta d\eta }{2\pi \mu_{0}\left[{\left(\left|x-\eta \right|\right)}^{3}+{\left(y-a-b+\delta b/h\right)}^{3}+{\left(z-\delta \right)}^{3}\right]}\left(\begin{array}{c} \left|x-\eta \right|\\ y-a-b+\delta b/h\\ z-\delta \end{array}\right) \end{array}\right..$$

The tangential component *B_y_* of the magnetic flux leakage signal intensity can be obtained by integrating the defect depth as shown in Equation (2):

(2) $$B_{y}=\int_{-c}^{c}d\eta \int_{0}^{h}dB_{1y}+\int_{-c}^{c}d\eta \int_{0}^{h}dB_{2y}.$$

**Figure 6:**
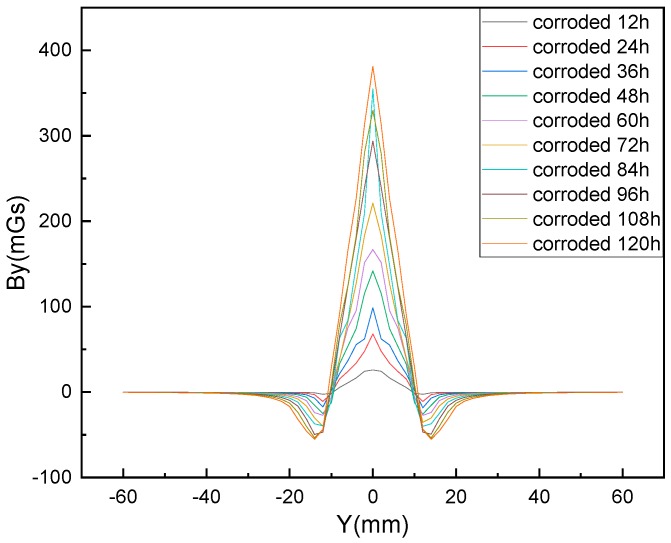
Schematic calculation of the *B_y_* component of the magnetic flux leakage signal along the *y*-axis direction under various corrosion conditions.

The test parameters are set according to the actual parameters of the corrosion test. The parameters of test piece 1 are selected, and using a MATLAB simulation calculation, we obtain a variation of the tangential component *B_y_* of the magnetic flux leakage signal to change along the *y*-axis direction, as shown in Figure 6. It can be seen that the magnetic signal has a sudden change in the rusted area, and the strength of the magnetic signal increases with the increase of the rust time.

## 3. Test Conclusions and Discussion

### 3.1. Magnetic Leakage Signal of Concrete Specimen

**Figure 7:**
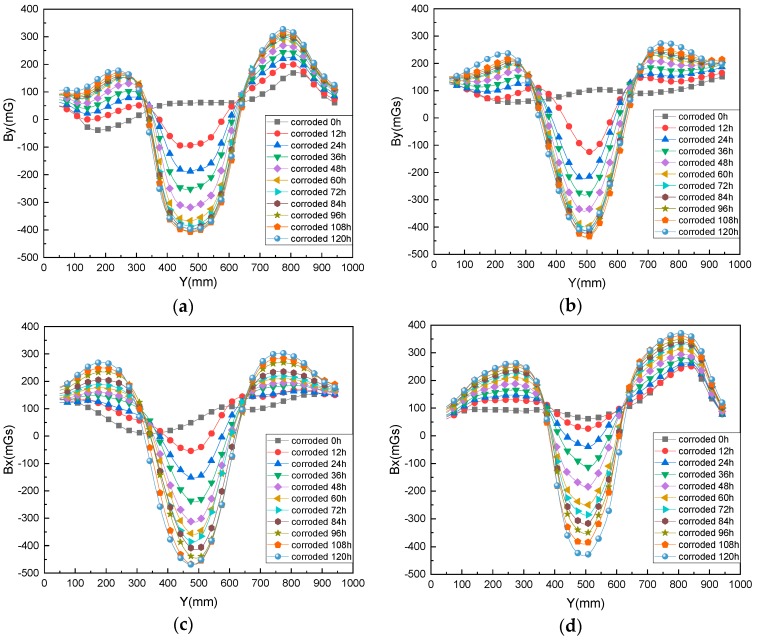
Variation of magnetic signal *B_y_* during rusting of test piece under various rust conditions as (**a**) test piece 1; (**b**) test piece 2; (**c**) test piece 3; and (**d**) test piece 4.

Since the pictures are similar, only reinforced concrete specimens 1–4 are subjected to each rusting condition, and the tangential component *B_y_* of the magnetic direction of the reinforcing bar is compared—that is, the *Y-B_y_* distribution curve, as shown in Figure 7. It can be seen that after each test piece is rusted, a magnetic flux leakage signal is generated at the position of the target rust area, forming a distinct trough in the center position of the target rust area. Moreover, the valley of the magnetic flux leakage signal deepens as the rust time increases. The width between the two intersection points of each group of *Y-B_y_* curves is the width of the actual rust area. As the degree of rust is deepened, the wave width of the *Y-B_y_* distribution curve is also widened simultaneously, indicating that the rusted area is gradually expanding. This is because the electrolyte spreads to the surrounding area in the concrete. The electrolyte further spreads along the crack to both sides when the concrete protective layer is cracked by the rusting force generated through the accumulation of the rust product, resulting in corrosion in the part outside the target area. Therefore, the width of the magnetic flux leakage signal distribution in the corroded area gradually increases.

### 3.2. The Relationship between the Change of Magnetic Flux Leakage Signal and the Diameter of the Steel Bar of Steel-Reinforced Concrete Specimens

#### 3.2.1. Selection of Origin of the Magnetic Flux Leakage Signal

The comparison of the extreme values of the leakage magnetic signals under various rust conditions is not enough to reflect all the positions and working conditions, especially the leakage magnetic signals under the uncorroded conditions. Therefore, the magnetic flux leakage signals are compared, and the “magnetic flux signal origin” (*B_y_*_0_, *Y*_0_) can be defined as a reference point for all magnetic signal curves to facilitate comparison of the magnetic flux leakage signals under all operating conditions.

**Figure 8:**
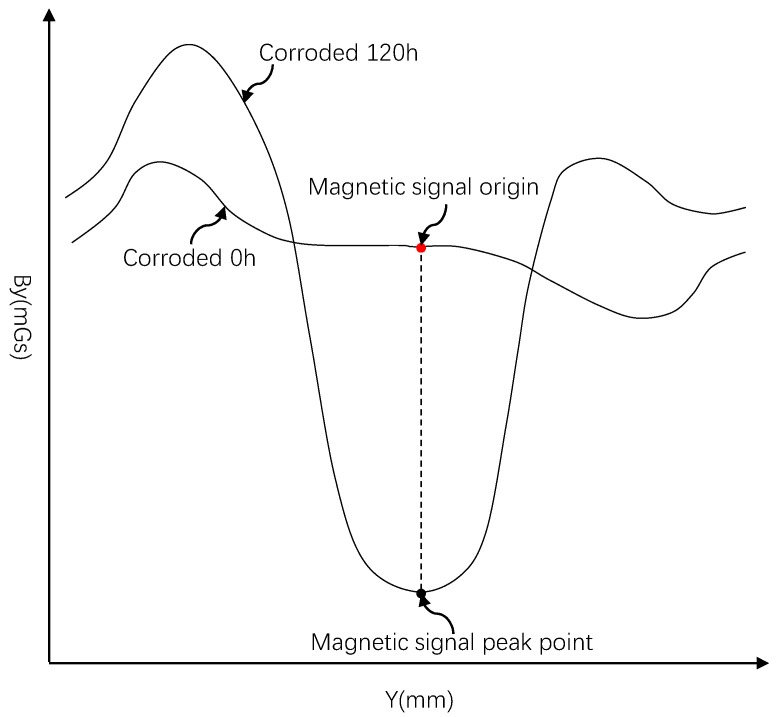
Schematic diagram of magnetic signal origin selection.

The valley extreme points of the magnetic flux leakage signal curve corresponding to the target rust area under each rust condition are selected. For the same test piece, the valley extreme value of the magnetic flux leakage signal curve of the rusted area under each rust condition is at the same *Y* coordinate. Therefore, it is only necessary to find the peak value of the valley in the rust 120 h condition to obtain the *Y* coordinate of the extreme point of each rust phase. Define the point on the magnetic flux leakage signal curve at 0 h corresponding to this coordinate as the “origin of magnetic flux leakage signal” (*B_y_*_0_, *Y*_0_), as shown in Figure 8.

#### 3.2.2. Proposal of Rust-Magnetic Fluctuation Parameter Mc

The “rust-magnetic fluctuation parameter” *Mc* needs to be defined in order to further study the relationship between the extreme value of the tangential component of the leakage magnetic flux generated by the corrosion of the steel-reinforced concrete specimens and the influence factors, as shown in Equations (3) and (4):

(3) $$S_{q}=\int_{y_{\min}}^{y_{\max}}\left|B_{y}-B_{y0}\right|dy,$$

(4) $$Mc=\frac{S_{q\max}-S_{q\min}}{S_{q\min}}=\frac{\int_{y_{\min}}^{y_{\max}}\left|B_{y\max}-B_{y0}\right|dy-\int_{y_{\min}}^{y_{\max}}\left|B_{y\min}-B_{y0}\right|dy}{\int_{y_{\min}}^{y_{\max}}\left|B_{y\min}-B_{y0}\right|dy},$$

where *B_y_*_max_ is the *B_y_* value under each rust condition, *B_y_*_min_ is the *B_y_* value under the uncorroded condition, *S_q_* is the area enclosed by the magnetic flux leakage signal curve under all rust conditions, and the origin of the rust origin *B*_*y*0_ extended straight line direction *B_y_* = *B_y_*_0_. The ratio of *S_q_*_max_ under rust condition to *S_q_*_min_ under the uncorroded condition and then to rust-free phase *S_q_*_min_ is the rust-magnetic fluctuation parameter *Mc*. The faster the rate of change of the *Mc* value, the greater the fluctuation of the *B_y_* value in the tangential direction of the magnetic flux leakage signal curve—that is, the more severe the corrosion.

#### 3.2.3. Correlation between Rust-Magnetic Fluctuation Parameter Mc and Steel Bar Diameter

**Figure 9:**
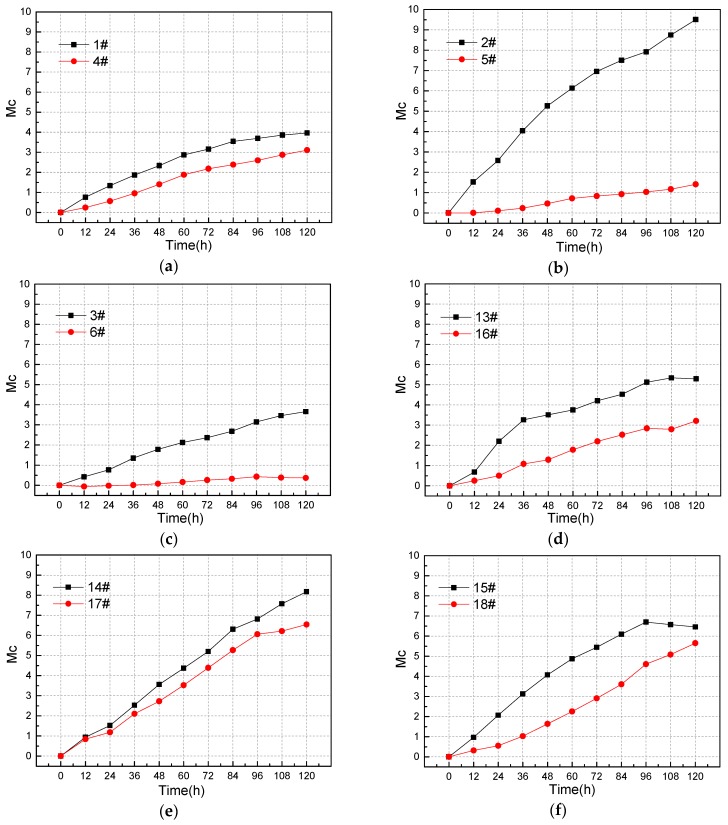

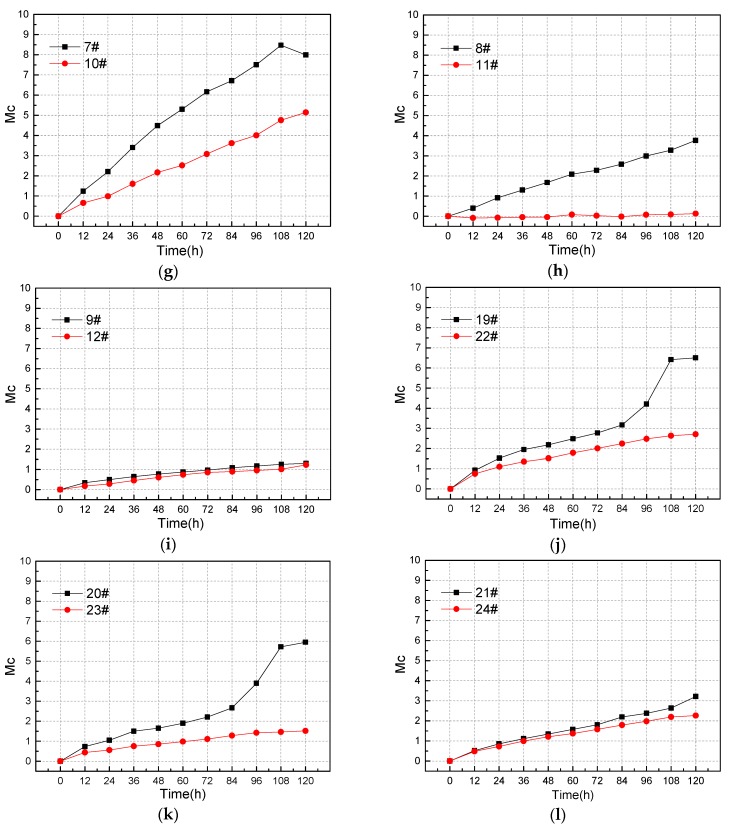
Mc-Time curve comparing the diameter of the steel bar.

Figure 9 is the *Mc-time* curve where the abscissa is the rust time, with *h* as the unit. The ordinate is the rust-magnetic fluctuation parameter *Mc*, where Mc is a constant and has no unit. A black curve represents a test piece with a diameter of 12 mm and a red curve represents a test piece with a diameter of 16 mm. According to Faraday’s first law of electrolysis, it can be expressed as Equation (5),

(5) $$\Delta W=\frac{M}{nF}I\Delta t,$$

where Δ*t* is the rust time, Δ*W* is the amount of corrosion of the metal in Δ*t*, *M* is the molar mass of Fe, and *M* = 56 g/mol; *n* is the number of electrons lost by Fe during oxidation, where *n* = 2; *F* is a constant Faraday, 1*F* = 96,485 C; *I* is the current flowing out of the anode; the rust degree *C* of the steel bar is expressed by the section loss rate, as shown in Equations (6) and (7).

(6) $$C=\frac{\Delta w}{W},$$

(7) $$W=\frac{\pi d^{2}l\rho }{4},$$

where *d* is the diameter of the steel bar, *l* is the width of the rusted area, and *ρ* = 7800 g/mm^3^. After calculation, the corrosion rates under the rust width of 100 mm and 150 mm are obtained, as shown in Table 3 and Table 4.

It can be seen from Table 3 that the theoretical time point for fracture of the 12 mm diameter steel bar is between 84 h and 96 h when the rust width is 100 mm. After the steel bar is broken due to corrosion, the magnetic field will suddenly change at the rusted position. Therefore, near 84 h and 96 h, the leakage magnetic field of the 12 mm specimen will abruptly change. It can be found from the Figure 9g–l that the slopes of the *Mc-Time* curves of specimens 13–15 and 19–21 all have mutations near 84 h and 96 h. Thus, the sudden change of the magnetic flux leakage signal is accurately reflected.

For the steel bars of different diameters, the loss of the cross-section of the steel bars is the same, and the rust degree is different under the same rust time. Therefore, the smaller the diameter of the steel bars, the larger the proportion of the diameter reduction; the more the magnetic charge concentrated above the steel bars, the stronger the magnetic flux leakage signal. Specifically, under the same rust time, the smaller the diameter of the reinforcing bar, the larger the trough extreme value generated by the tangential component B_y_ of the magnetic flux leakage signal and the larger the overall fluctuation of the linear curve of the magnetic flux leakage signal. As shown in Figure 9a–l, it is obvious that the rate of the *Mc* parameter change of the steel-reinforced concrete specimen with a steel bar diameter of 12 mm is larger compared to the steel bar with a diameter of 16 mm. Under the same working conditions, the *Mc* parameter is more sensitive to the specimen with a smaller diameter of the reinforcing bar. In other words, the smaller the diameter of the steel, the greater the rate of change of *Mc* parameters.

To sum up, the change and abrupt change of the magnetic flux leakage signal of the steel bar can be accurately reflected by the *Mc* parameter, as well as the influence of the diameter of the steel bar on the change of the magnetic flux leakage signal. Besides, the *Mc* parameters can be quantitatively analyzed.

## 4. Conclusions

In this paper, we sought to study the relationship between the rate of change of magnetic flux leakage signals on the surface of corroded steel bars as the degree of corrosion deepens and the diameter of the bar. Reinforced steel-clad concrete specimens with different diameters of rebars were used as research objects. The electrochemical principle was used to accelerate the corrosion of steel bars, and the self-designed three-dimensional magnetic signal acquisition device was used to perform the bar scan test on the steel-clad concrete samples under different corrosion conditions.

The conclusions are drawn as follows:

(1) In the steel bar corrosion test of the steel-reinforced concrete test piece, the tangential component *B_y_* of the magnetic flux leakage signal on the surface of the test piece is deepened as the degree of corrosion increases; the extreme value in the rusted area becomes larger and larger. The width of the rusted area becomes wider as the degree of rust increases due to the diffusion of the electrolyte in the concrete. The “origin of magnetic flux leakage signal” *B_y_*_0_ is defined as a reference point in the data analysis in order to carry out data comparison more finely; it can more effectively add the magnetic signal curve under each rust condition to the comparison.
(2) Besides, the “rust magnetic fluctuation parameter” *Mc* is also proposed in this paper. Let the area sandwiched between the magnetic signal curve and the straight line *B_y_* = *B_y_*_0_ formed by the magnetic signal origin extending in the direction of the reinforcing bar under each corrosion condition be *S_q_*_max_. Let the area sandwiched by the magnetic signal curve and *B_y_* = *B_y_*_0_ under the non-corrosive condition be *S_q_*_min_. (*S_q_*_max_ − *S_q_*_min_)/*S_q_*_min_ is the fluctuation parameter *Mc*.
(3) The sudden change of the leakage magnetic field caused by the rust of the steel bar can be accurately reflected by the *Mc* parameter as well as the rate of change of the tangential component *B_y_* of the magnetic flux leakage signal with the deepening of the rust depth. Moreover, it can be connected to various influencing factors. In this paper, the coefficient is related to the diameter of the steel bar. It is verified that under the same rust condition, the smaller the diameter of the steel bar, the larger the valley extreme value of the magnetic flux leakage signal, that is, the greater the fluctuation.
(4) Based on the spontaneous magnetic leakage theory, a new idea and data processing method for the non-destructive detection of steel corrosion is provided by metal spontaneous magnetic leakage is this study. We were able to effectively remove the effect of the diameter of the reinforcing bar on the magnetic flux leakage signal. Furthermore, the accuracy of non-destructive detection of steel bar corrosion by the spontaneous magnetic leakage technology is effectively improved. The method has low cost, simple equipment, and safe and convenient operation, as well as good application prospects.

## Figures and Tables

**Table 1:** Chemical composition of the rebar (mass fraction).

Type of Steel	Chemical Composition				
	C	Si	Mn	P	S
**HRB400**	0.2	0.4	1.3	0.03	0.02

**Table 2:** Number and naming of steel-reinforced concrete specimens.

Number	Name	Number	Name
1#	2000-120-30-a	13#	2000-120-30-b
2#	2000-120-40-a	14#	2000-120-40-b
3#	2000-120-50-a	15#	2000-120-50-b
4#	2000-160-30-a	16#	2000-160-30-b
5#	2000-160-40-a	17#	2000-160-40-b
6#	2000-160-50-a	18#	2000-160-50-b
7#	1500-120-30-a	19#	1500-120-30-b
8#	1500-120-40-a	20#	1500-120-40-b
9#	1500-120-50-a	21#	1500-120-50-b
10#	1500-160-30-a	22#	1500-160-30-b
11#	1500-160-40-a	23#	1500-160-40-b
12#	1500-160-50-a	24#	1500-160-50-b

**Table 3:** Calculation of corrosion rate with corrosion width of 100 mm.

Rebar Diameter	12 h	24 h	36 h	48 h	60 h	72 h	84 h	96 h	108 h	120 h
***d* = 12 mm**	14.2%	28.4%	42.5%	56.7%	70.9%	85.1%	99.3%	113.4%	127.6%	141.8%
***d* = 16 mm**	8.0%	16.0%	23.9%	31.9%	39.9%	47.9%	55.8%	63.8%	71.8%	79.8%

**Table 4:** Calculation of corrosion rate with corrosion width of 150 mm.

Rebar Diameter	12 h	24 h	36 h	48 h	60 h	72 h	84 h	96 h	108 h	120 h
***d* = 12 mm**	9.5%	18.9%	28.4%	37.8%	47.3%	56.7%	66.2%	75.6%	85.1%	94.5%
***d* = 16 mm**	5.3%	10.6%	16.0%	21.3%	26.6%	31.9%	37.2%	42.5%	47.9%	53.2%

## References

[1] Li L. (2018). Corrosion Monitoring of Reinforced Concrete Structure Based on Electrochemical Theory. J. Nanoelectron. Optoelectron..

[2] Jung J.S., Lee B.Y., Lee K.S. (2019). Experimental Study on the Structural Performance Degradation of Corrosion-Damaged Reinforced Concrete Beams. Adv. Civ. Eng..

[3] James A., Bazarchi E., Chiniforush A.A., Aghdam P.P., Hosseini M.R., Akbarnezhad A., Martek I., Ghodoosi F. (2019). Rebar Corrosion Detection, Protection, and Rehabilitation of Reinforced Concrete Structures in Coastal Environments: A Review. Constr. Build. Mater..

[4] Zhang H., Liao L., Zhao R., Zhou J., Yang M., Xia R. (2016). The Non-Destructive Test of Steel Corrosion in Reinforced Concrete Bridges Using a Micro-Magnetic Sensor. Sensors.

[5] Zhang H., Zhou J., Zhao R., Liao L., Yang M., Xia R. (2017). Experimental Study On Detection of Rebar Corrosion in Concrete Based On Metal Magnetic Memory. Int. J. Robot. Autom..

[6] Shi X.M., Zhu J.S. Study on characteristics of local stress concentration of corroded reinforcing bars in concrete. Proceedings of the 20th National Bridge Conference.

[7] Sassine E., Laurens S., Francois R., Ringot E. (2018). A Critical Discussion on Rebar Electrical Continuity and Usual Interpretation Thresholds in the Field of Half-Cell Potential Measurements in Steel Reinforced Concrete. Mater. Struct..

[8] Scully J.R. (2000). Polarization Resistance Method for Determination of Instantaneous Corrosion Rates. Corrosion.

[9] Angst U., Buechler M. (2015). On the Applicability of the Stern-Geary Relationship to Determine Instantaneous Corrosion Rates in Macro-Cell Corrosion. Mater. Corros..

[10] Gonzalez J.A., Cobo A., Gonzalez M.N., Feliu S. (2001). On-Site Determination of Corrosion Rate in Reinforced Concrete Structures by Use of Galvanostatic Pulses. Corros. Sci..

[11] Macdonald D.D., Mckubre M., Urquidimacdonald M. (1988). Theoretical Assessment of Ac Impedance Spectroscopy for Detecting Corrosion of Rebar in Reinforced-Concrete. Corrosion.

[12] Bellezze T., Giuliani G., Roventi G. (2018). Study of Stainless Steels Corrosion in a Strong Acid Mixture. Part 1: Cyclic Potentiodynamic Polarization Curves Examined by Means of an Analytical Method. Corros. Sci..

[13] Zou X., Schmitt T., Perloff D., Wu N., Yu T., Wang X. (2015). Nondestructive Corrosion Detection Using Fiber Optic Photoacoustic Ultrasound Generator. Measurement.

[14] Du C., Twumasi J.O., Tang Q., Guo X., Zhou J., Yu T., Wang X. (2018). All-Optical Photoacoustic Sensors for Steel Rebar Corrosion Monitoring. Sensors.

[15] Cho H.M., Cho H.S., Kim K.S., Lim H.W., Park S.Y., Lee S.R., Kim K.C., Je U.K., Park Y.O., Hong D.K. (2015). Experimental Study On the Application of a Compressed-Sensing (CS)-based Deblurring Method in X-Ray Nondestructive Testing and its Image Performance. NDT&E Int..

[16] Hanke R., Fuchs T., Uhlmann N. (2008). X-Ray Based Methods for Non-Destructive Testing and Material Characterization. Nucl. Instrum. Methods A.

[17] Shim H., Choi M.S., Lee D.H., Hur D.H. (2016). A Prediction Method for the General Corrosion Behavior of Alloy 690 Steam Generator Tube Using Eddy Current Testing. Nucl. Eng. Des..

[18] Xu C., Zhou N., Xie J., Gong X., Chen G., Song G. (2016). Investigation On Eddy Current Pulsed Thermography to Detect Hidden Cracks On Corroded Metal Surface. NDT&E Int..

[19] Wicker M., Alduse B.P., Jung S. (2018). Detection of Hidden Corrosion in Metal Roofing Shingles Utilizing Infrared Thermography. J. Build. Eng..

[20] Ruiko W., Toshiaki M. (2017). Study On Evaluation of Corrosion Condition of Reinforcing Bar Embedded Concrete Using Infrared Thermal Imaging Camera. Int. Soc. Opt. Photonics.

[21] Li D., Tan M., Zhang S., Ou J. (2018). Stress Corrosion Damage Evolution Analysis and Mechanism Identification for Prestressed Steel Strands Using Acoustic Emission Technique. Struct. Control Health Monit..

[22] Mangual J., ElBatanouny M.K., Velez W., Ziehl P., Matta F., Gonzalez M. (2012). Characterization of Corrosion Damage in Prestressed Concrete Using Acoustic Emission. Int. Soc. Opt. Photonics.

[23] Qu Y.H., Zhang H., Zhao R.Q., Liao L., Zhou Y. (2019). Research On the Method of Predicting Corrosion Width of Cables Based On the Spontaneous Magnetic Flux Leakage. Materials.

[24] Liu B., Fu Y., Xu B. (2015). Study on Metal Magnetic Memory Testing Mechanism. Res. Nondestruct. Eval..

[25] Guo P.J., Guan W.H., Yan C.Z., Qin Z.C., Chen X.D. (2015). Characteristics of Spontaneous Magnetic Field of Artificial Crack Induced by Complex Stress in Large Components. Amsterdam.

[26] Xia R., Zhou J., Zhang H., Zhou D., Zhang Z. (2019). Experimental Study On Corrosion of Unstressed Steel Strand Based On Metal Magnetic Memory. KSCE J. Civ. Eng..

